# Alternative meiotic chromatid segregation in the holocentric plant *Luzula elegans*

**DOI:** 10.1038/ncomms5979

**Published:** 2014-10-08

**Authors:** Stefan Heckmann, Maja Jankowska, Veit Schubert, Katrin Kumke, Wei Ma, Andreas Houben

**Affiliations:** 1 Department of Cytogenetics and Genome Analysis, Leibniz Institute of Plant Genetics and Crop Plant Research (IPK), Corrensstraße 3, 06466 Gatersleben, Germany

## Abstract

Holocentric chromosomes occur in a number of independent eukaryotic lineages. They form holokinetic kinetochores along the entire poleward chromatid surfaces, and owing to this alternative chromosome structure, species with holocentric chromosomes cannot use the two-step loss of cohesion during meiosis typical for monocentric chromosomes. Here we show that the plant *Luzula elegans* maintains a holocentric chromosome architecture and behaviour throughout meiosis, and in contrast to monopolar sister centromere orientation, the unfused holokinetic sister centromeres behave as two distinct functional units during meiosis I, resulting in sister chromatid separation. Homologous non-sister chromatids remain terminally linked after metaphase I, by satellite DNA*-*enriched chromatin threads, until metaphase II. They then separate at anaphase II. Thus, an inverted sequence of meiotic sister chromatid segregation occurs. This alternative meiotic process is most likely one possible adaptation to handle a holocentric chromosome architecture and behaviour during meiosis.

## Abstract (web summary)

Conventionally, meiosis depends on a two-step loss of chromosome cohesion that requires distinction between chromosome arms and sister centromeres. Heckmann *et al.* show that a plant that lacks a single defined centromere overcomes this problem by inverting the sequence of meiotic segregation events.

## Graphical abstract

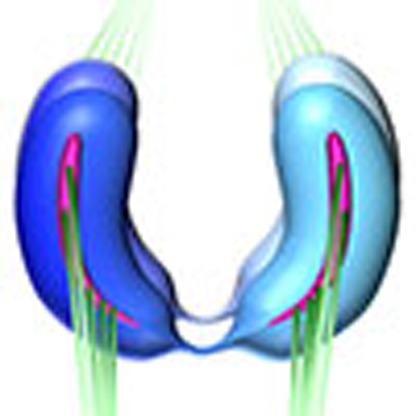

Meiosis is a key event for stable sexual reproduction using a unique form of a cell division to generate haploid recombined gametes. It comprises one round of DNA replication followed by two rounds of chromosome segregation. During meiotic divisions in species with monocentric chromosomes cohesion between sister chromatids is released in two steps: (i) along chromosome arms during meiosis I and (ii) at sister centromeres during meiosis II (ref. 1). At metaphase I, sister chromatids attach via fused sister kinetochores to the meiotic spindle and mono-orient, allowing homologous chromosome segregation (reductional division), whereas at metaphase II sister chromatids bi-orient and segregate equationally in a mitosis-like manner (Fig. 1a).

Holocentric chromosomes are found in certain independent eukaryotic lineages^2^,^3^,^4^,^5^,^6^,^7^. They lack a primary constriction and form holokinetic kinetochores distributed along almost the entire poleward surface of the chromatids, except at their ends. Despite divergent chromosome architectures, kinetochore components and strategies to enable mitotic chromosome segregation are similar for species with mono- and holocentric chromosomes^2^,^8^,^9^. However, owing to their alternative chromosome structure, holocentrics cannot rely on the two-step loss of cohesion during meiosis that is typical for monocentrics. As an adaptation, holocentrics commonly restrict the crossover frequency per homologue to one or two distal-to-terminal chiasmata^10^,^11^,^12^,^13^.

The following three principle options exist to deal with holocentricity during meiosis: (i) ‘chromosome remodelling’, (ii) ‘functional monocentricity’ and (iii) ‘inverted chromatid segregation’. In the first case (Fig. 1b,c), owing to one off-centred crossover^13^, a cruciform bivalent with a short (mid-bivalent) and a long arm is formed in the nematode *Caenorhabditis elegans*^14^. The crossover location determines which chromosome end orientates polewards^14^,^15^ and it triggers a spatial distribution of various kinetochore proteins to distinct chromosomal domains^16^,^17^,^18^,^19^,^20^,^21^. This facilitates the two-step loss of cohesion^18^,^20^,^21^; at the mid-bivalent during meiosis I enabling separation of homologues and at the long arm during meiosis II enabling separation of sisters. In oocytes, it also establishes the primary kinetic force, a pushing from the mid-bivalent for kinetochore-independent homologue separation during meiosis I (Fig. 1b). This reductional division is accompanied by lateral microtubule bundles sheathing the bivalent, and by an encasement of homologous sisters by outer kinetochore proteins^16^,^17^,^19^. However, in spermatocytes, microtubule bundles are enriched at the bivalent ends facing polewards (Fig. 1c)^17^,^22^, reminiscent of a ‘telokinetic-like’ behaviour (Fig. 1d).

In the second case (Fig. 1d), holocentric chromosomes act like monocentric chromosomes, that is, microtubules attach only to a restricted terminal chromosomal region (‘telokinetic behaviour’), as found in different *Heteroptera* and *Parascaris*^11^,^23^,^24^,^25^,^26^. Thus, a separation of homologues during meiosis I and of sister chromatids during meiosis II occurs. Which chromosome end acquires kinetic activity during the first meiotic division is random^11^. Interestingly, the chromosome end showing no kinetic activity during meiosis I can acquire kinetic activity during meiosis II (ref. 11). In metaphase II chromosomes of *Triatoma infestans*, active sister kinetochores can even be found at opposite termini (Fig. 1d)^23^.

In the third case (Fig. 1e), holocentric bivalents align at metaphase I in such a manner that homologous sister chromatids face opposite poles and separate from each other, that is, non-sister chromatids of homologous chromosomes segregate together at anaphase I. By metaphase II, homologous non-sister chromatids are joined together and then separate from each other at anaphase II. Thus, an inverted sequence of chromatid segregation events in contrast to the typical sequence observed in species with monocentric chromosomes occurs. This type of meiosis has been postulated for both plants and animals for example, for the wood rush *Luzula elegans* (formerly *L. purpurea*)^27^,^28^,^29^,^30^ and the mealybug *Planococcus citri*^31^.

By direct visualization of the dynamics of meiotic centromere behaviour and of the pattern of chromosome segregation, we provide proof of our hypothesis that the chromosomes of *L. elegans* are structurally and functionally holocentric throughout meiosis, and that an inverted sequence of sister chromatid segregation occurs during meiosis. Furthermore, we propose that terminal satellite DNA repeat-enriched chromatin threads assist the pairwise movement and the linkage of homologous non-sister chromatids up to metaphase II to enable the faithful formation of haploid gametes.

## Results

### Holokinetic sister centromeres remain unfused at metaphase I

The centromere-specific histone H3 variant CENH3 (also called CENP-A) marks the active centromeres of mono- as well as holocentric chromosomes^2^,^8^,^32^. Mitotic chromosomes of *Luzula* species display a defined longitudinal CENH3-positive centromeric groove along each sister chromatid, except at the chromosome ends^33^,^34^. To evaluate whether this holocentric chromosome structure is maintained during meiosis antibodies specific for CENH3 of *Luzula*^33^,^34^, the centromere-located Thr120-phosphorylated variant of histone H2A^35^,^36^,^37^ and α-tubulin were used.

During early pachytene, CENH3 immunosignals are dispersed and form numerous small foci (Fig. 2a). When the three homologous chromosome pairs are discernible at diplotene, and at the subsequent diakinesis, two parallel dotted lines of CENH3 signals are visible per homologous chromosome (Fig. 2b,c). Two predominant bivalent configurations are apparent (Fig. 2); of 289 analysed bivalents, 43.0% and 47.3% displayed a rod- and ring-like structure, respectively. 9.7% of all analysed bivalents represented a cruciform-like structure reminiscent of the bivalent structure found in *C. elegans*^38^.

At metaphase I, CENH3 and H2AThr120ph immunolabelling revealed four distinct linear centromeres per bivalent, reflecting two pairs of individual sister centromeres (Fig. 2d,e). These are the centromeres of the four homologous chromatids to which CENH3, H2AThr120ph and the ends of α-tubulin localize (Fig. 2f and Supplementary Movie 1). The attachment sites to the meiotic microtubules are distributed along the whole-longitudinal sister centromeres in a similar way as to how they are organized at mitosis^33^,^34^. Thus, unlike a monopolar centromere orientation of homologous chromosomes, bipolar-orientated holokinetic sister centromeres of *L. elegans* behave as two distinct functional units at meiosis I.

As described by Nordenskiold^28^, one or two preferentially terminal chiasmata per bivalent occurred. One terminal (rod bivalent), one interstitial (cruciform bivalent) and two terminal (ring bivalent) chiasmata were found (Supplementary Table 1). Eight telomere signals per bivalent, two at the end of each homologous chromosome and four in the middle, reflect an end-to-end association of homologous chromosomes (Fig. 3a,b). Note that a 4′,6-diamidino-2-phenylindole (DAPI)-positive region distal to the *Arabidopsis*-type telomere repeats was found within the end-to-end associated regions of homologous chromosomes forming rod and ring bivalents. Thus, the telomeres are not localized at the morphological chromosome termini and seem to be folded back (Fig. 3b). Terminal localization of telomeres was confirmed by the analysis of extended pachytene chromosomes (Supplementary Fig. 1).

To address the question of whether both ends of a given chromosome can form an end-to-end association, LeSAT28- and LeSAT63-specific hybridization probes were used to allow the discrimination of chromosomes and of chromosome ends within one bivalent (Fig. 3c). Each of the different homologous chromosome pairs can form a ring, a rod or a cruciform bivalent with no obvious preference regarding the occurrence of one or the other configuration existing between the bivalents (Fig. 3d–f and Supplementary Table 1). Each end of a given chromosome can take part in the end-to-end association (Fig. 3g,h).

### An inverted sequence of meiotic sister chromatid segregation

To verify the process of ‘inverted meiosis’ proposed for several species with holocentric chromosomes, including *Luzula*^27^,^28^,^29^,^30^,^39^, the segregation events of chromatids during gamete formation were studied in detail. Similar to species with monocentric chromosomes, bivalents align at the metaphase I plate perpendicular to the spindle axis (Fig. 2f). A bipolar microtubule attachment to the two holocentric sister kinetochores per homologous chromosome and a monopolar microtubule attachment to the associated homologous non-sister chromatids was found (Figs 2f and 4a). Thus, in contrast to monocentric chromosome species, sister chromatids divide equationally and homologous non-sister chromatids migrate together to the same pole at anaphase I. A connection between one or both ends was detected for 95% of the analysed homologous non-sister chromatids during anaphase I (Fig. 4b and Supplementary Table 2).

At the end of anaphase I, the region between separated chromatids is filled with a large number of microtubules (Fig. 4c), most likely as a prerequisite for phragmoplast formation. During telophase I decondensation progresses, the tubulin bundles disappear, the CENH3 immunosignals diffuse and the chromatids of both homologues stay associated. Finally, diffused chromatin with CENH3 foci comprising chromatids within the two distinctive ‘interkinesis-like’ nuclei is present (Fig. 4d). At the onset of the second meiotic division, homologous non-sister chromatids are still linked. Condensed associated holocentric chromatids align at the metaphase II plate perpendicular to the spindle axis (Fig. 4e). Chromatin threads between homologous non-sister chromatids are still visible at metaphase II (Fig. 4f) for 57% of associated homologous non-sister chromatids (Supplementary Table 2). During anaphase II, individual chromatids segregate holokinetically in a mitosis-like manner (Fig. 4g) and four haploid gametes are formed (Fig. 4h). Despite a reversal in the order of meiotic segregation events, analysis of tetrads with chromosome-specific markers LeSAT7 and LeSAT28 (Fig. 3c) revealed the formation of haploid gametes (Fig. 4h).

To verify the claimed inverse order of chromatid segregation events, meiocytes of a heterozygous plant possessing an X-ray-induced chromosome break were studied by fluorescence *in situ* hybridization (FISH). Breakage of one chromosome of pair 1 resulted in two chromosome fragments of different sizes (Fig. 5a). As described for *Luzula* species^27^,^28^,^29^, holocentric chromosome fragments were also stably transmitted in *L. elegans*. The observed mirror image (at both poles, the same chromatids are present) of all chromosomes, including chromosome fragments during anaphase I, provides further evidence that the sister chromatids separate already during meiosis I (Fig. 5b–d). The long chromosome fragment of pair 1 pairs with the unbroken homologous chromosome, while the remaining small chromosome fragment (sf) is unpaired at metaphase I. Owing to the separation of sister chromatids and the end-to-end connection of homologous non-sister chromatids, chromosome pair 1 forms unequally sized end-to-end connected non-sister chromatids at anaphase I. The chromatids of the sf also divide. Hence, the sister chromatids divide first during meiosis I and the homologous non-sister chromatids then divide next during meiosis II. Thus, an inverted sequence of sister chromatid segregation occurs. A similar sequence of meiotic segregation events as schematically summarized in Fig. 6 was observed for the holocentric chromosomes of *L. luzuloides* (Supplementary Fig. 2).

### Chromatin threads connect homologous non-sister chromatids

We observed that homologous non-sister chromatids forming rod bivalents are connected to each other by chromatin threads during meiosis I. These are <0.5 μm thick and up to 6-μm long (Fig. 7a). Comparable interchromatid connections were not observed in mitotic cells. To reveal their composition, we asked whether preferential terminal-enriched satellite repeats colocalize with these threads. LeSAT7- and LeSAT-11-specific hybridization probes showed that these repetitive sequences are enriched in the chromatin threads (Fig. 7a–c). Notably, satellite LeSAT7 seems to be localized distal to the *Arabidopsis*-type telomeres both in metaphase I and II chromosomes (Figs 4b and 7b), similar to the observed DAPI-positive chromosome ends distal to the *Arabidopsis*-type telomeres. We therefore suspect that these terminal regions loop back to facilitate the observed internal telomere localization, end-to-end associations and to result in the morphological subterminal telomere positions.

In contrast to MI chromosomes of *C. elegans*^18^,^40^, we did not observe any signal specific for the serine 10 or serine 28 phosphorylated histone H3 in the region restricted to the short arm of the bivalent. This so-called mid-bivalent region in *C. elegans* is distal to the chiasma, where cohesion will be removed during the first meiotic division^18^,^40^. Instead, *L. elegans* exhibits a uniform distribution of these post-translational modifications of histone H3 (Supplementary Fig. 3).

### Cytologically prophase I is conventional in *L. elegans*

To test whether meiotic processes during prophase I differ between holo- and monocentric species, the synaptonemal complex and axis formation was examined and it was found that the distributions of ASY1- and ZYP1-immunolabelling signals were similar to those described for species with monocentric chromosomes^41^,^42^. Polymerized Asy1-stretches appear during leptotene/zygotene and linear-like Zyp1-signals during zygotene/pachytene along the bivalents (Fig. 8a,b).

To decipher whether telomeres cluster in a bouquet configuration, as in many species with monocentric chromosomes^43^, the distribution of *Arabidopsis*-type telomeres and subterminal LeSAT7 satellite DNA repeats was studied by FISH during different prophase I stages (Fig. 9). In premeiotic nuclei, both signal types are distributed throughout the nucleus (Fig. 9a). At the onset of leptotene, the number of distinct LeSAT7 signal foci reduces, until all subtelomeric LeSAT7 signals cluster at zygotene (Fig. 9b,c). This suggests that all three chromosome pairs are synapsed. Finally, the separation of paired homologous chromosomes (desynapsis) appears to occur from the chromosome ends during (late) pachytene (Fig. 9d). Thus, in contrast to *C. elegans*, a typical zygotene telomere-mediated bouquet-like configuration of chromosomes takes place, as suggested by the clustering of all LeSAT7 subterminal satellite DNA and the *Arabidopsis*-type telomere-specific hybridization signals in one nuclear hemisphere. A similar, bouquet-like configuration was also observed in the closely related species *L. luzuloides*, as indicated by clustering of telomeres at zygotene (Supplementary Fig. 4).

## Discussion

**Figure 1:**
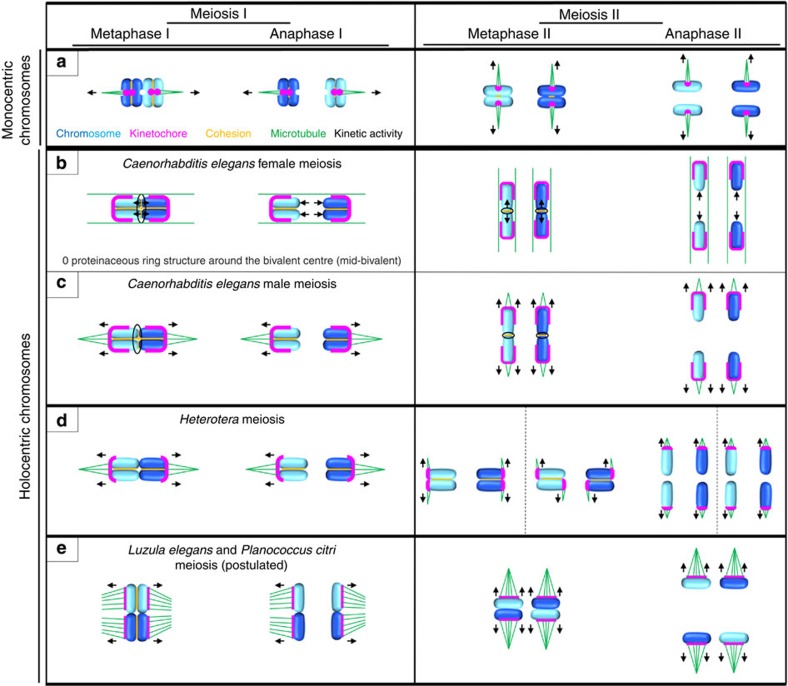
Schematic model of meiosis in species with monocentric chromosomes and of meiotic adaptations in species with holocentric chromosomes. (**a**) Monocentric chromosomes: Sister chromatids mono-orient at metaphase I via fused sister kinetochores, allowing homologous chromosome segregation, whereas at metaphase II sister chromatids bi-orient and segregate from each other. Note a ring bivalent configuration of metacentric chromosomes. (**b**–**e**) Holocentric chromosomes: Several options exist to deal with a holocentric chromosome architecture and meiosis: (**b**,**c**) chromosome remodelling, (**d**) functional monocentric chromosomes and (**e**) ‘inverted chromatid segregation’. Note that rod-shaped bivalents are shown (**b**–**e**), recombination events are not indicated (**a**–**e**) and sister chromatids are of the same colour. (**b**,**c**) One typically off-centred crossover leads to a cruciform bivalent with a short and a long arm. Owing to progressive condensation, bivalents occur ‘rod-shaped’ at metaphase I (short and long arms are not indicated in **b**,**c**). The crossover location triggers a distinct spatiotemporal protein distribution, for example, proteins including Aurora B or chromokinesin KLP-19 form a ring around the mid-bivalent, and (outer) kinetochore proteins encase each homologue. This distribution conditions cohesion loss at the mid-bivalent and retention at long arms during anaphase I enabling homologue separation. During meiosis II, proteins including KLP-19 surround the ring-shaped sister chromatid interface while (outer) kinetochore proteins encase individual sisters. At anaphase II, cohesion gets lost at the sister chromatid interface allowing sister chromatid separation. (**b**) KLP-19 mediates a pushing force from the mid-bivalent supported by lateral microtubules ensheathing bivalents during meiosis I and linked sister chromatids during meiosis II. (**c**) Bivalent ends facing polewards are attached by microtubules. (**d**) Microtubule attachment to a restricted terminal chromosomal region. Holocentric chromosomes become functional monocentric (‘telokinetic‘) enabling separation of homologues during meiosis I and of sister chromatids during meiosis II. Active sister kinetochores can form even at opposite metaphase II chromosome termini. (**e**) Homologous sister chromatids, attached by microtubules along nearly their entire lengths, face opposite poles during metaphase I and separate from each other at anaphase I. Homologous non-sister chromatids are joined by metaphase II and separate at anaphase II. Hence, an inverted meiotic chromatid segregation sequence compared to the typical meiotic segregation pattern in monocentric chromosome species occurs.

**Figure 2:**
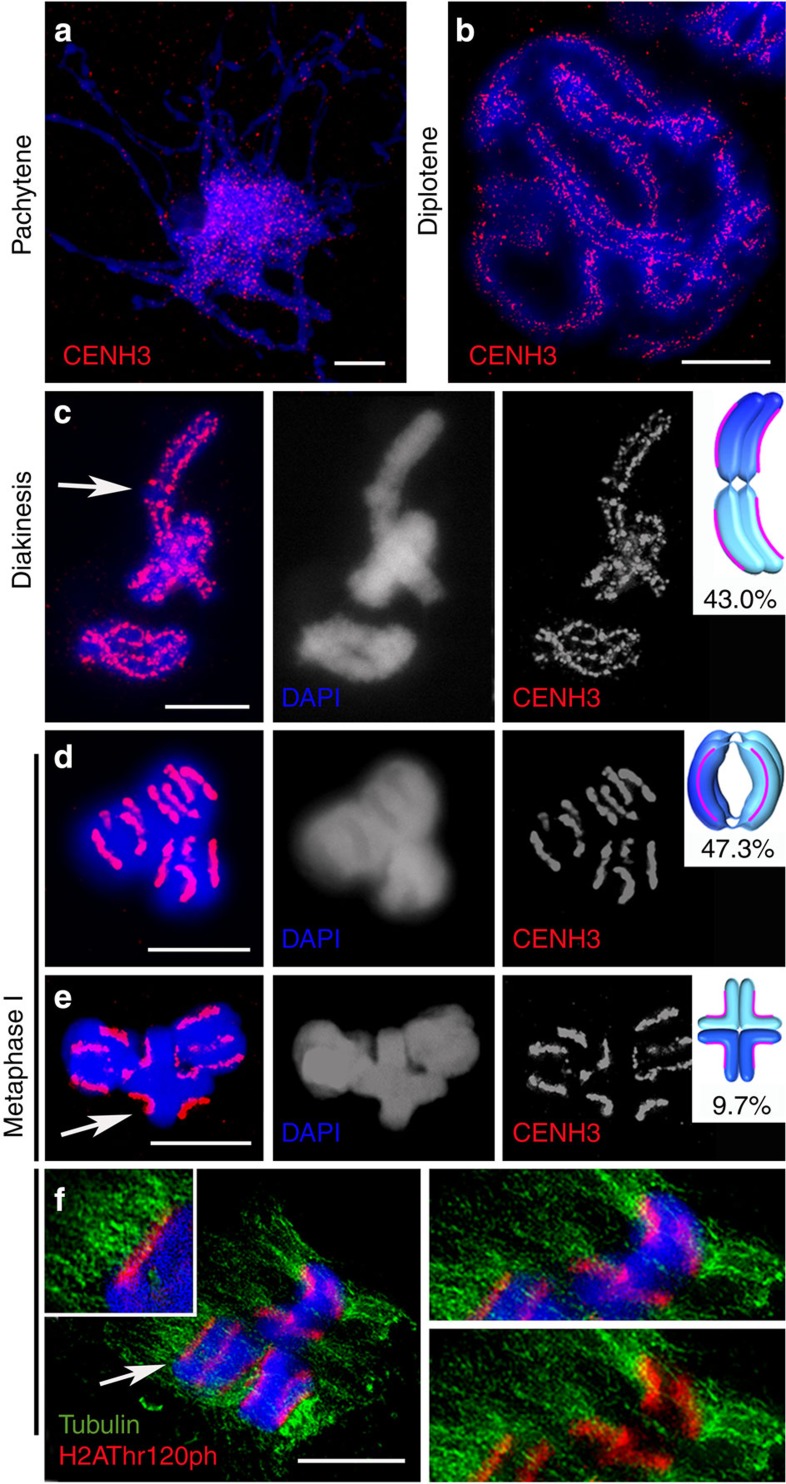
Holokinetic sister centromeres of *L. elegans* remain separated entailing bipolar spindle attachment at metaphase I. Early pachytene (**a**), diplotene (**b**), diakinesis (**c**) and metaphase I (**d**–**f**). Rod (arrowed in **c**), ring (**d**) and cruciform (arrowed in **e**) bivalent configurations after detection of CENH3 (red) by immunolabelling. Arrow in **c** shows the CENH3-free end-to-end association region of the homologous chromosomes. Schemata illustrate bivalent configurations. Centromeres are shown as red lines and the sister chromatids are depicted in the same colour. Recombination events are not indicated. Frequencies of configurations are based on 289 analysed bivalents. Metaphase I (**f**) after immunodetection of H2AThr120ph (red) and α-tubulin (green). Inset (left) shows enlarged spindle microtubules attachment along the entire length of the holocentric centromere. The right images show a further enlarged U-shaped rod-bivalent. DNA counterstained with DAPI (blue). Scale bars, 10 μm.

**Figure 3:**
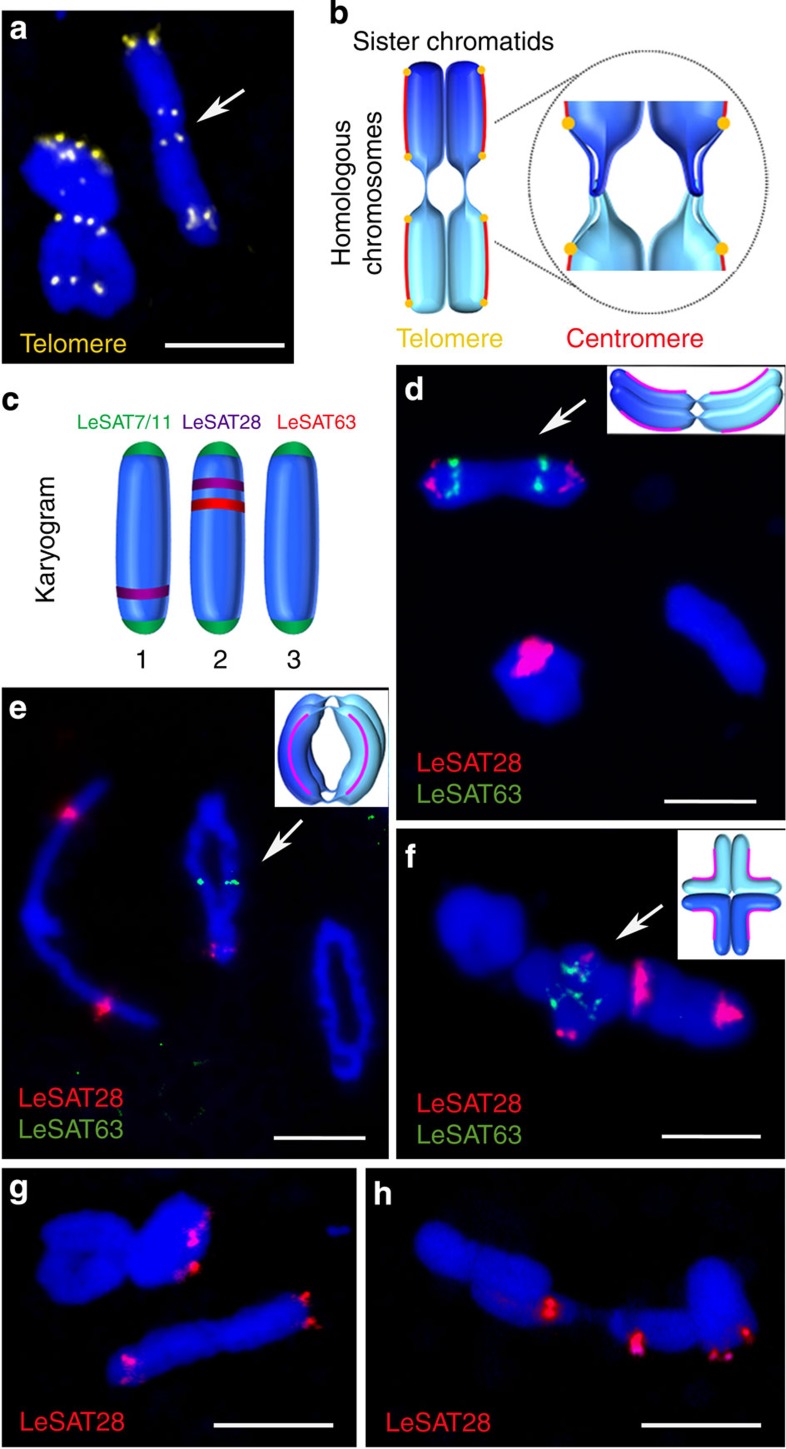
Various bivalent configurations occur during metaphase I. (**a**) Eight distinct telomere signals emerge both in ring and rod bivalents after FISH with an *Arabidopsis*-type-specific telomere probe (yellow). Arrow indicates an end-to-end association between homologous chromosomes of a rod bivalent. A DAPI-positive region between telomeres of homologues is involved in the end-to-end association. (**b**) Model of an end-to-end associated rod bivalent. Inset shows the proposed structure of the interchromatid link. Telomeric regions loop back to facilitate the observed internal telomere localization and end-to-end association between homologous non-sister chromatids. (**c**) Karyogram of *L. elegans* depicting the distribution of utilized satellite repeats. (**d**–**f**) Each homologous chromosomes pair can form a ring, rod or cruciform-like bivalent configuration as shown by FISH with LeSAT28- (red) and LeSAT63-specific (green) probes. (**g**,**h**) Each chromosome end can mediate the end-to-end association with its homologous partner (46 and 54% of LeSAT28-negative or -positive chromosome ends, respectively, were found in 50 analysed rod bivalents). Chromosomes were counterstained with DAPI (blue). Scale bars, 10 μm.

**Figure 4:**
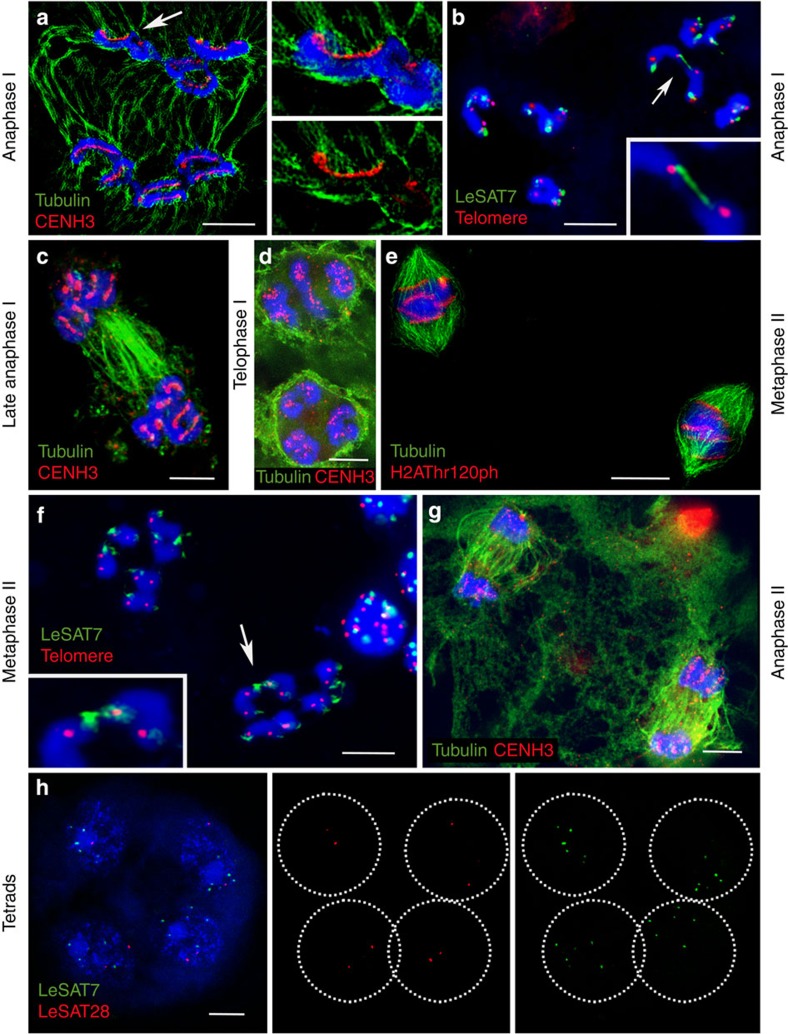
Sister chromatids divide first and homologous non-sister chromatids separate afterwards. Anaphase I (**a**–**c**), telophase I (**d**), metaphase II (**e**,**f**) and anaphase II (**g**) chromosomes immunolabelled with centromere- (CENH3 or H2AThr120ph; red) and α-tubulin-specific (green) antibodies. The right images in **a** show enlarged spindle microtubules attachment along the entire length of the holocentric chromatids. Distribution of subterminal LeSAT7 (green) and telomere (red) repeats during anaphase I (**b**) and metaphase II (**f**), as well as of chromosome-specific markers (LeSAT7 (green) and LeSAT28 (red)) in tetrads (**h**) after FISH. The insets and arrows in **b**,**f** show LeSAT7-enriched chromatin threads connecting two homologous non-sister chromatids. Equal number of LeSAT7 and LeSAT28 signals in each tetrad nucleus indicates proper haploidization (six signals of LeSAT7 and two signals of LeSAT28 per tetrad nucleus). DNA counterstained with DAPI (blue). Scale bars, 10 μm.

**Figure 5:**
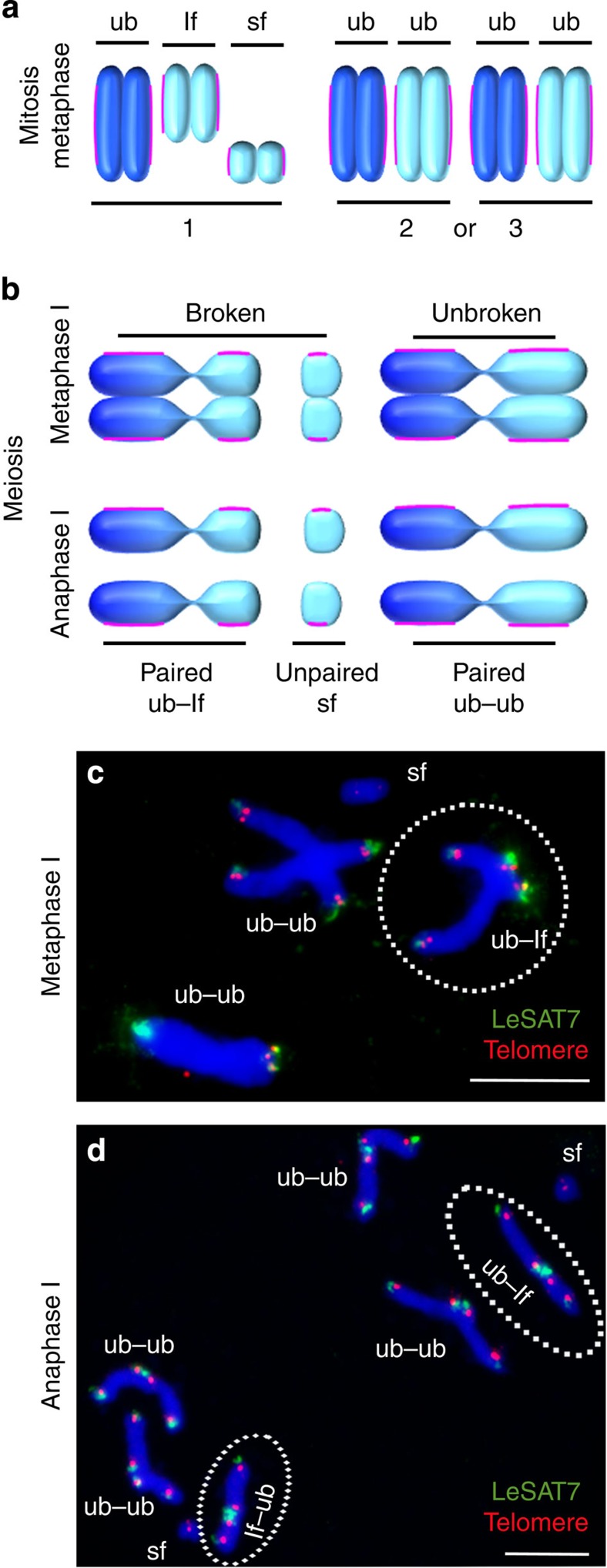
A heterozygote plant with an unequally sized chromosome pair 1 shows the separation of sister chromatids during meiosis I. (**a**) Schemata of mitotic metaphase chromosomes in a plant carrying two chromosome fragments of different size (lf, long fragment; sf, short fragment; ub, unbroken homologous chromosome) as a result of the breakage of one homologue of chromosome pair 1. (**b**) Schemata of the segregation behaviour of unbroken (ub) and broken (lf and sf) chromosomes during meta- and anaphase I, respectively. (**c**,**d**) FISH with LeSAT7 (green) and telomere (red) probes identifies a single-chromosome breakage event resulting in a large and a small chromosome fragment. (**b**,**c**) The large fragment forms an end-to-end association with its homologous partner due to chromosome pairing (encircled), whereas the small fragment remains unpaired. (**b**,**d**) Balanced separation of heterozygous chromatids of pair 1 that form unequally sized end-to-end connected homologous non-sister chromatids (ub–lf, encircled) at anaphase I illustrates that sister chromatids are separated and not homologues. The chromatids of the sf divide, too. Note, due to ‘telomere healing’, fragmented chromosomes also possess telomeres. DNA counterstained with DAPI (blue). Scale bars, 10 μm.

The data presented here demonstrate striking differences between the meiotic process and kinetochore geometry of species with mono- and holocentric chromosomes. We provide evidence that the chromosomes of *L. elegans* and of *L. luzuloides* are structurally and functionally holocentric during all stages of meiosis. Other than in the case of *C. elegans*, which undergoes chromosome remodelling during meiosis (Fig. 1b,c)^38^, and where during female meiosis even a kinetochore-independent chromosome segregation mechanism takes place (Fig. 1b)^16^,^17^,^19^, the meiotic centromere organization has not been investigated in other species with holocentric chromosomes.

Similar to mitosis, during both meiotic division stages, a longitudinal centromere along each chromatid, except at their ends, containing CENH3 and H2AThr120phos was found, to which α-tubulin fibres attach. It is likely that multiple centromeric subunits (foci in early prophase nuclei) fuse to a single functional linear-like centromere during meiotic divisions, as suggested for mitotic chromosomes^2^,^44^.

In contrast to monocentric chromosomes^1^, sister centromeres do not fuse during meiosis I; therefore, unfused sister centromeres result in bipolar attachment of microtubules at metaphase I and the separation of sister chromatids occurs. Thus, the mechanism that regulates kinetochore geometry in meiosis I differs between mono- and holocentric chromosome species. Similarly, mutations of genes involved in the fusion of sister kinetochores, such as MIS12 (ref. 45) or REC8 (ref. 46), and in the protection of centromeric cohesion during meiosis I, such as shugoshin^47^,^48^, also result in unfused centromeres and premature separation of sister chromatids at meiosis I in species with monocentric chromosomes.

**Figure 6:**
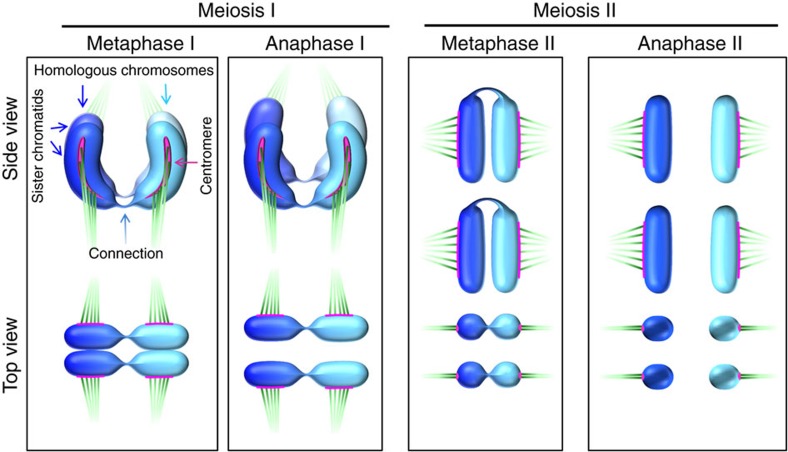
Model illustrating the structure of meiotic chromosomes and the sequence of meiotic segregation events in *L. elegans*. The chromosomes are shown from the side and the top view (with respect to the metaphase plate). Holocentric U-shaped bivalents (homologous chromosomes are end-to-end associated) align at metaphase I in such a manner that sister chromatids rather than homologous chromosomes are separated during meiosis I. Homologous non-sister chromatids are terminally linked by satellite DNA-enriched chromatin threads until metaphase II to ensure faithful transmission of holocentric chromatids. During meiosis II, the associated homologous non-sister chromatids become holokinetically separated. Therefore, an inverted sequence of meiotic events from the cytological point of view takes place. From the top view, the bivalents appear typically rod-like, whereas from the side view they occur mainly U-shaped.

**Figure 7:**
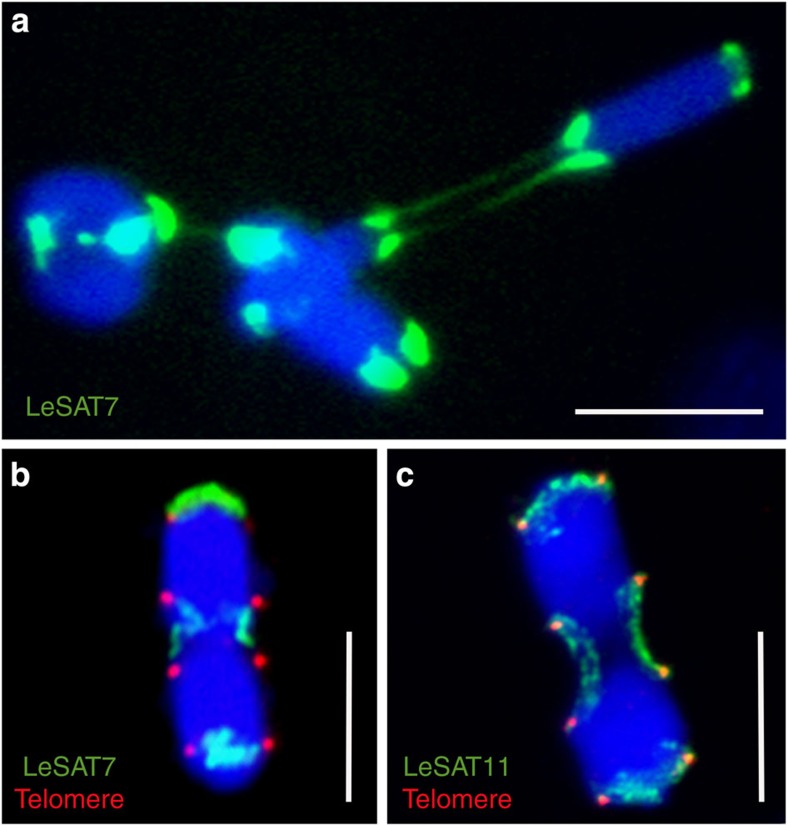
Satellite DNA-enriched chromatin threads connect homologous non-sister chromatids terminally. Satellites LeSAT7 (**a**,**b**) and LeSAT11 (**c**) mediate the end-to-end association of homologous chromosomes during meiosis I. Heterochromatic fibres differ in length between homologous partner chromosomes and cells. Note distal location of LeSAT7 compared with the telomeres (**b**). The bivalents were labelled with LeSAT7 (green), LeSAT11 (green) and *Arabidopsis*-type telomere (red) probes, and counterstained with DAPI (blue). Scale bars, 10 μm.

**Figure 8:**
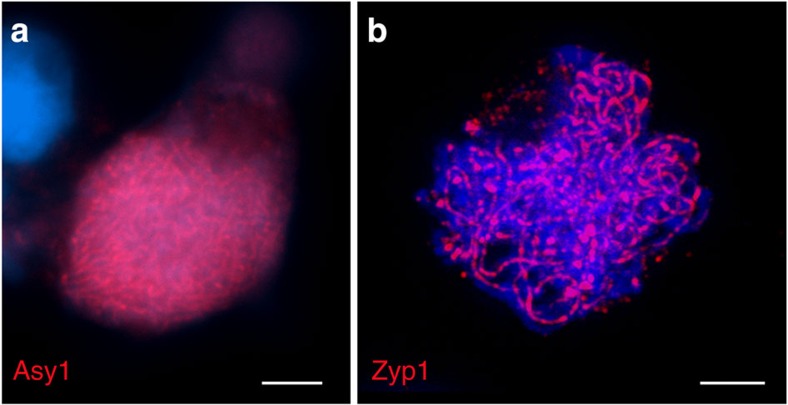
Synaptonemal complex and axis formation occurs during prophase I in *L. elegans*. Detection of ASY1 during leptotene/zygotene (**a**; red) and of ZYP1 during zygotene/pachytene (**b**; red) by immunolabelling. DNA counterstained with DAPI (blue). Scale bars, 10 μm.

**Figure 9:**
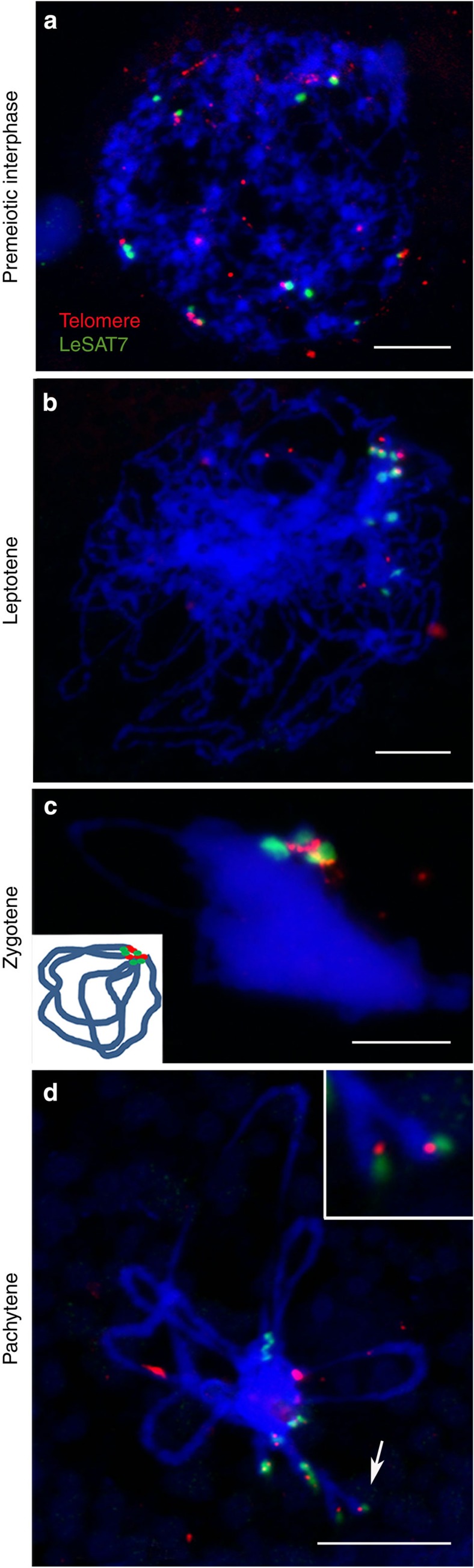
Holocentric *L. elegans* chromosomes form a bouquet-like configuration during prophase I. (**a**) Premeiotic interphase, (**b**) leptotene, (**c**) zygotene and (**d**) pachytene cells labelled with the subterminal satellite DNA LeSAT7 (green) and telomere (red) probes by FISH. DNA counterstained with DAPI (blue). (**c**) Note a zygotene-typical bouquet-like configuration as indicated by clustering of LeSAT7 and telomere signals in one nuclear hemisphere; schematically shown by the inserted model, too. Inset and arrow (**d**) show the onset of desynapsis at the end of paired homologous chromosomes during pachytene.

In order to deal with holocentricity, and the unfused sister centromeres during meiosis I, *Luzula* performs an alternative sequence of chromatid segregation events (Fig. 6). Whereas in monocentric chromosomes sister chromatid cohesion is released in a two-step manner (first along chromosome arms during meiosis I and second at centromeres during meiosis II; Fig. 1a)^1^, sister chromatid cohesion in *Luzula* is released along the whole holocentric chromosomes during metaphase I. The observed U-shape of the rod bivalents at the metaphase/anaphase I transition is most likely mediated by spindle forces comparable to the sickle shape of mitotic chromosomes at metaphase/anaphase transition^33^.

Homologous chromosomes preferentially form either a rod or a ring bivalent at metaphase I. One or two terminal chiasmata persists, or possibly chiasma frequency might be skewed towards the chromosome end as described^49^. However, taking into account that early prophase I events (when crossovers are formed) are similar to those in species with monocentric chromosomes (for example, axis, synaptonemal complex and telomere bouquet formation), it is likely that crossovers are established along the chromosomes, as suggested for other supposed meiotically holocentric chromosome species, for example, in *Heteroptera*^11^,^50^. Finally, only terminal chiasmata may persist by being released later than those in interstitial regions. In some relatively rare cases, a single interstitial crossover might persist, which is visible as a cruciform bivalent. Alternatively, ‘sticky’ terminal heterochromatin may enable an achiasmatic end-to-end association of homologous chromosomes and may link the homologous non-sister chromatids (see also refs 2, 6, 31). If terminal chiasmata persist until metaphase I, or are resolved after metaphase I until telophase I, then an achiasmatic cohesin mediated mechanism could facilitate the association of homologous non-sister chromatids until metaphase II.

It is noteworthy that no active centromeres are formed in these terminal chromosomal regions in species with holocentric chromosomes^33^,^34^,^51^,^52^ that are crossover-enriched, reminiscent of the reduced crossover frequency in centromeric as compared with non-centromeric regions of species with monocentric chromosomes, such as *Arabidopsis thaliana*^53^. Possibly, in both chromosome types, crossovers occur preferentially in non-centromeric regions.

How do homologous non-sister chromatids that divide in meiosis I remain associated long enough to segregate in meiosis II? We propose that the observed chromatin threads assist the pairwise transport and joining of homologous non-sister chromatids until anaphase II. Achiasmatic heterochromatin threads, found during *Drosophila* oocyte meiosis^54^ and spermatogenesis in the crane-fly^55^, point to a more widespread occurrence of such chromatin threads between segregating partner chromosomes. The observed threads in *L. elegans* are not caused by catenated late replicating DNA, as homologous and non-sister chromatids are connected. FISH showed that different terminal satellite DNAs, cytologically distal to telomere repeats, are involved in the end-to-end association of the homologous chromosomes during meiosis I and of the homologous non-sister chromatids from metaphase I until metaphase II. Moreover, as repetitive DNA enriched in (sub-)subterminal centromere-free regions is a common feature in holocentric plant and animal autosomes (reviewed in refs 2, 44), it is likely that subterminal satellite DNAs or heterochromatin, in general, might be needed to deal with holocentricity during meiosis. Beside satellite DNA-enriched heterochromatin, cohesin might specifically localize to this distinct subterminal chromatin and assist the end-to-end association.

In conclusion, we demonstrate that the chromosomes of *L. elegans* are functional holocentric throughout meiosis and perform an inverted sequence of chromatid segregation events. We propose that terminal satellite DNA repeat-enriched chromatin threads assist the correct joining of homologous non-sister chromatids enabling faithful formation of haploid gametes.

## Methods

### Plant material and culture conditions

Seeds of *L. elegans* (Lowe) (2*n*=6) (Vouchers at the Herbarium Gatersleben: GAT 7,852–7,856) and *L. luzuloides* (Lam.) (2*n*=12) (kindly provided by the Botanical Garden of the Martin-Luther-University, Halle–Wittenberg) were germinated on wet filter paper under long-day conditions (16 h light/8 h dark, 20 °C/18 °C). The plantlets were then transferred to soil and cultivated for 6–8 weeks under short-day conditions (8 h light/16 h dark, 20 °C/18 °C). Finally, the plants were transferred for at least 3 month to vernalizing conditions (10 h light/14 h dark, 4 °C) and then returned to long-day conditions (13 h light/11 h dark, 20 °C/16 °C) to induce flowering.

### Indirect immunolabelling

Flower buds were fixed for 45 min in ice-cold 4% (w/v) paraformaldehyde either in 1 × PBS (pH 7.4) or in 1 × microtubules stabilizing buffer (MTSB) buffer (50 mM PIPES, 5 mM MgSO_4_ and 5 mM EGTA, pH 7.2) to preserve microtubules. All following steps were carried out with the same buffer used for fixation, that is, either PBS or MTSB. After washing in 1 × PBS/MTSB, meiotic chromosome spreads were prepared by squashing. Immunolabelling was performed as described^56^. The following dilutions of primary antibodies were used: 1:100 of a rabbit anti-LnCENH3 (ref. 34), 1:200 of a monoclonal mouse anti-α-tubulin (clone DM 1A, Sigma), 1:250 of a rabbit anti-Zyp1 (ref. 42), 1:250 of a rabbit anti-Asy1 (ref. 57), 1:200 of a anti-H2A120phos (Abcam, ab111492), 1:100 of a rabbit anti-H3S28phos (Millipore, 07–145) and 1:300 of a monoclonal mouse anti-H3S10ph (Abcam, ab14955) antibody. A Cy3-conjugated anti-rabbit IgG (Dianova) and a fluorescein isothiocyanate-conjugated anti-mouse Alexa 488 antibody (Molecular Probes), each at a 1:400 dilution, were used as secondary antibodies.

### Probe preparation and fluorescence *in situ* hybridization

Chromosomes were identified with the satellite repeats LeSAT7, 11, 28 and 63 according to ref. 44. The telomeres were detected using the PCR-generated *Arabidopsis*-type telomere repeat according to ref. 58. Meiotic chromosome spreads of macerated anthers were prepared from ethanol–acetic acid (3:1, v/v)-fixed flower buds according to ref. 59 with minor modifications. Before slide preparation, the fixed material was washed three times for 5 min each with H_2_O and with 10 mM citric acid–sodium buffer (citrate buffer, pH 4.6). Maceration was done in 0.1% (w/v) each of cytohelicase, pectolyase and cellulase in 10 mM citrate buffer (pH 4.6) at 37 °C for 30 min and stopped by washing two times for 5 min each in citrate buffer and in H_2_O. Anthers were placed onto a slide in a drop of ice-cold 60% acetic acid and dispersed with a metal needle. Ice-cold acetic acid (60%) was added to the cell suspension, mixed with a needle and kept for 2 min at room temperature. Once more, ice-cold 60% acetic acid was added and the slide was placed on a hot plate (45 °C) for 2 min. During this time, the drop was moved by the needle. The slide was removed from the hot plate and freshly prepared ice-cold ethanol–acetic acid (3:1, v/v) was added in a circle around the suspension. After a few seconds, the suspension was discarded and more ice-cold ethanol–acetic acid was added to the slide. The slide was placed in 60% acetic acid for 10 min, briefly immersed in 96% ethanol and then air dried. After spreading, the specimens were washed twice for 5 min in 2 × Saline-Sodium Citrate buffer (SSC), treated with 0.1% pepsin in 1 N HCl for 6 min at 37 °C and washed twice in 2 × SSC for 5 min each. Post fixation was performed for 10 min in 2.5% (w/v) formaldehyde in 2 × SSC, pH 8.0. Further, the slides were washed in 2 × SSC three times for 5 min, dehydrated in an ethanol series and air dried. FISH was performed according to ref. 44.

### Microscopy

Wide-field fluorescence microscopic images were recorded with an Olympus BX61 microscope (Olympus; http://www.olympus.com) equipped with an ORCA-ER CCD camera (Hamamatsu; http://www.hamamatsu.com). Three-dimensional (3D) deconvolution was applied to reduce the out-of-focus blur. Image stacks of 10–11 slices per specimen were acquired, and the maximum intensity projections were processed with the programme AnalySIS (Soft Imaging System; http://www.soft-imaging.net). Grey scale images were pseudo-coloured with Adobe Photoshop. To achieve an optical resolution of ~120 nm, structured illumination microscopy was applied using a C-Apo 63 × /1.2 W Korr objective of an Elyra microscope system (Zeiss; http://www.zeiss.com). Structured illumination microscopy image stacks were used to produce 3D movies by the ZEN software (Zeiss).

## Author contributions

A.H. contributed to project planning and design; V.S. conducted super-resolution light microscopy; S.H., M.J., V.S., K.K., W.M. and A.H. analysed the data; and S.H. and A.H. wrote the manuscript.

## Additional information

**How to cite this article**: Heckmann, S. *et al.* Alternative meiotic chromatid segregation in the holocentric plant *Luzula elegans*. *Nat. Commun.* 5:4979 doi: 10.1038/ncomms5979 (2014).

## Supplementary Material

Supplementary Figures and Tables Supplementary Figures 1-4 and Supplementary Tables 1-2

Supplementary Movie 1 Behaviour of L. elegans chromosomes during metaphase I. During metaphase I the L. elegans bivalents appear U-shaped due to spindle fibre attachment along the whole holokinetic centromeres. The centromeres are labelled by H2AThr120ph (red), the spindle fibres by α-tubulin (green) and chromatin with DAPI (blue).
