# Supplementary material for: Alternative meiotic chromatid segregation in the holocentric plant Luzula elegans
Source: Nat Commun. 2014 Oct 8;5:4979. doi: 10.1038/ncomms5979 (PMC4214429; doi:10.1038/ncomms5979)
Supplement: Supplementary Figures and Tables — Supplementary Figures 1-4 and Supplementary Tables 1-2 [file ncomms5979-s1.pdf]

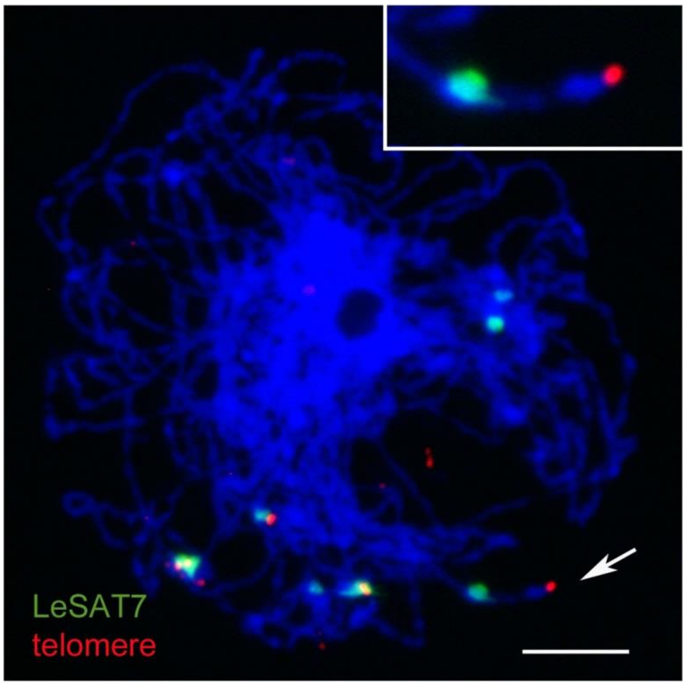

Supplementary Figure 1

***Arabidopsis*-type telomeres are localized at chromosome termini in close proximity to satellite DNA LeSAT7.** Extended pachytene chromosomes labelled with LeSAT7- (green) and telomere-specific (red) FISH probes. Inset and arrow show an enlarged chromosome end. DNA counterstained with DAPI (blue).Bar = 10 µm.

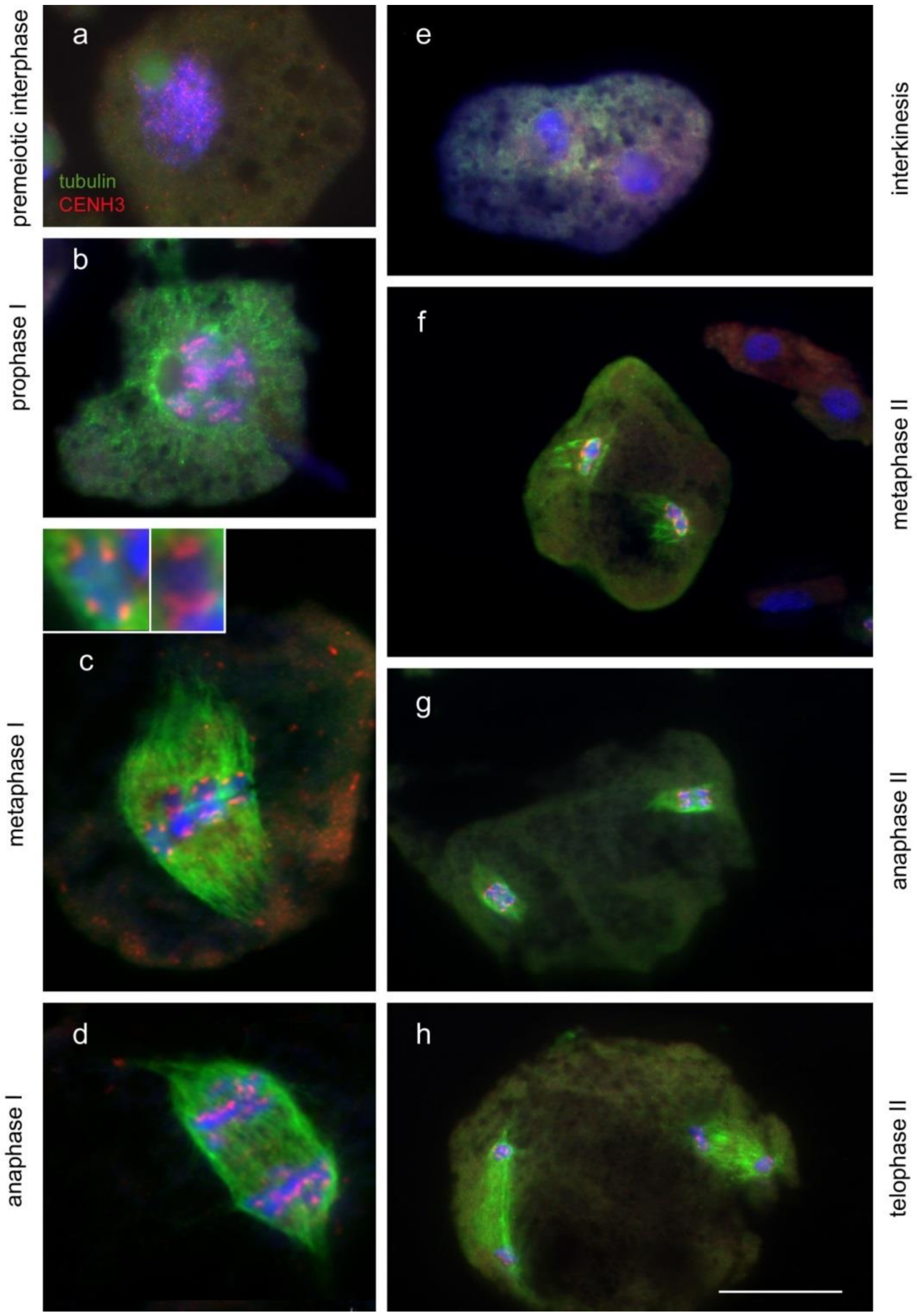

Supplementary Figure 2

Meiosis in *L. luzuloides*. A holocentric chromosome structure and an inverted order of chromatid segregation events were observed by immunostaining with anti-CENH3 (red) and anti-α-tubulin (green) antibodies. Insets in (c) show an enlarged cruciform bivalent and an enlarged rod bivalent with end-to-end associated homologous chromosomes. Nuclei were counterstained with DAPI (blue). Bar = 10 µm.

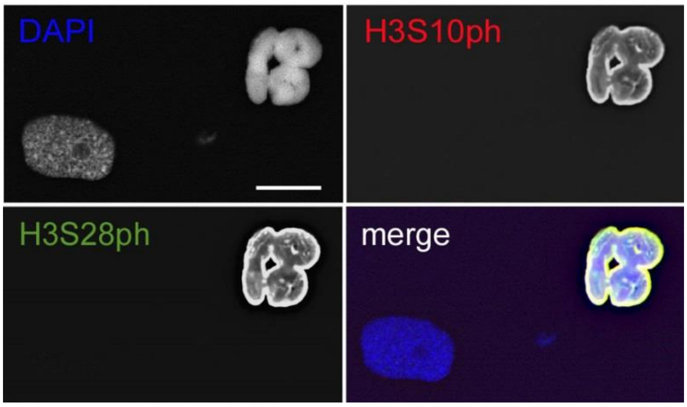

Supplementary Figure 3

***L. elegans* bivalents exhibit a uniform distribution of the cell cycle-dependent phosphorylation of histone H3 at serine 10 (H3S10ph; red) and serine 28 (H3S28ph; green) at metaphase I.** The entire chromosomes are H3 hyperphosphorylated. Nuclei were counterstained with DAPI (blue). Bar = 10 µm.

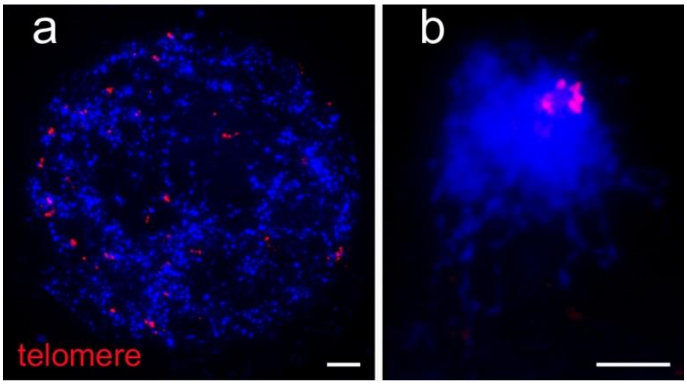

Supplementary Figure 4

Holocentric chromosomes of *L. luzuloides* behave in a similar way to monocentric chromosomes during prophase I. Telomeres were used as FISH probes. *Arabidopsis*-type telomere signals (red) are randomly distributed in the premeiotic nucleus (a). A bouquet-like configuration is present during zygotene; all telomere signals are clustered (b). Nuclei were counterstained with DAPI (blue). Bars = 10 µm.

Supplementary Table 1.

Chromosome-specific frequency of bivalent configurations during meiosis I in *L. elegans.*

	ring bivalent	rod bivalent	cruciform bivalent
Chromosome 1<br>number analysed = 40	47.5%	40.0%	12.5%
Chromosome 2<br>number analysed = 46	43.5%	45.6%	10.9%
Chromosome 3<br>number analysed = 43	48.8%	44.2%	7.0%

Supplementary Table 2.

Frequency of end-to-end connections of homologous non-sister chromatids at anaphase I and at the onset of anaphase II. Representative chromatids are shown in inserts after FISH with LeSAT7- (green) and telomere-(red) specific probes. The DNA is DAPI-stained (blue).

**anaphase I**		**onset of anaphase II**	
one connection	two connections	one connection	separated
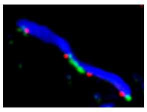	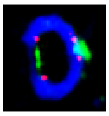	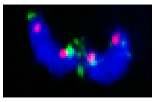	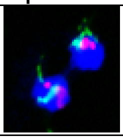
55%	45%	57%	43%
total number 200		total number 93	
